# Editorial

**Published:** 1838-02-14

**Authors:** 


THE
### MEDICAL EXAMINER.
PHILADELPHIA.
Wednesday, February 14, 1838.

In our Foreign Summary will be found a brief résumé of the remarks of M. Quetelet on the *Effects of Marriage*, taken from an able and elaborate analysis of his recent valuable work on *Man and the development of his Faculties*, in a late number of the Edinburgh Med. and Surg. Journal. Based as all his calculations are on the infallible and unfailing data of analysis and numbers, and his results being determined by the most cautious and vigilant induction, his opinions are entitled to consideration and respect. The especial importance of that part of the subject to which we have above alluded, viewed in both a moral and physical light, must, we think, be conceded by every one. Exercising so marked and vital an influence and control on the social state, marriage, too, is a point on which the practitioner of medicine is frequently consulted, and one on which it behooves him to have definite ideas and conceptions, as upon his knowledge, candour, and integrity the happiness of many may depend.

M. Quetelet has, to our mind, investigated this subject more philosophically and more satisfactorily than any one who has previously attempted it, because he has not suffered himself to be misled by the speciousness of theory and speculation, but has been contented with arriving at facts, and establishing truths by the laborious and tedious, but certain and stable process of statistical examination; and his conclusions are valuable because we know whence and how they were derived, and that they are undeniable. With us, however, M. Quetelet's work cannot be received as absolute authority, not from any inaccuracy on his part, but from the uncontrolable circumstance of modifying influences, which operate with us, and which must materially impair that entire aptness which alone can render results elsewhere obtained indisputable evidence; these are climate, manners, customs, difference of institutions, and what we believe to be a very prominent and important one, a purer moral atmosphere. But why should not similar investigations, pursued with the same rigid and scrutinizing truth, be conducted amongst us? Surely our capacities are equal to the task, and it would most abundantly repay him who should attempt it.

Our improved moral and social condition has been repeatedly attributed to the early age at which our marriages are in general contracted and consummated. Would it not be satisfactory to ascertain whether a vitiated and impaired physical state, with individual disease and national degeneracy, are not also entailed as a consequence? What too is the mental and bodily condition of the product of two immature beings, yet unfitted by nature for the great offices they have precipitately undertaken, compared to the offspring of vigor and perfection? These two questions are of such paramount importance to the well-being and good ordination of society, that they cannot too soon be satisfactorily resolved. We suspect that in all countries the result will eventually be found the same, and agreeable to that announced by M. Quetelet, and that a few years delay will bring at once improved and perfected functional requisites, as well as increased and more substantial worldly acquisitions, and consequently the chance ot greater ultimate happiness and prosperity. These remarks apply with peculiar force and truth to the female, the ill-effects of precocious maternity operating with especial prejudice upon her. The daily wrecks of beauty and loveliness in all the fulness of youth, are impressive and warning examples of the injurious influence of early marriages. With the male the reverse is probably correct: marriage with him is usually the goal and termination of excesses and dissipation, and the earnest of future temperance and moderation. We may hereafter again recur to this interesting topic, when Mr. Ryan's forthcoming work on "Marriage," comes under our notice.

We may remark here, that M. Quetelet is inclined to attribute, with justness we think, the frequent occurrence of still born children, which happens much more frequently in towns than in the country, to the absurd and ridiculous practice of *tight lacing*. This silly and criminal custom is persevered in by many women until the very completion of pregnancy, entailing the most deplorable consequences both on them and their offspring, with a pertinacity and hardihood almost incredible.

M. Quetelet in the course of his work gives several authentic statistical tables showing the proportion of illegitimate births, which seem on the continent of Europe to be frightfully increasing. In Paris we find that out of every 28 births 10 are illegitimate, exhibiting a state of moral terpitude and licentiousness appalling and unparaleled.

---

We announce with regret the death of Dr. Ansell W. Ives, of New York, after a protracted illness of intense suffering. He expired on Tuesday evening, the 6th inst., of Neuralgia, under which distressing affection he had been labouring for the past year. For the last nine months he endured the most excruciating and unspeakable agony, under which he eventually succumbed.

Dr. Ives was favourably known to the medical public as the Editor of the American edition of Paris' Pharmacologia, to which work he made a number of valuable additions.

---

Since the above was written we have received the melancholy tidings of the death of Dr. John Eberle, of Lexington, late of Cincinnati, after a protracted illness. He died on the 2nd inst. Dr. Eberle was well known as the author of "A Practice of Medicine," "An Elementary Treatise on Thrapeutics," with a number of other valuable works. He was originally we believe, from this state, and practised sometime in the interior of it. He resided in this city for several years, and was a Professor in the Jefferson Medical College..

